# Patient-derived small intestinal myofibroblasts direct perfused, physiologically responsive capillary development in a microfluidic Gut-on-a-Chip Model

**DOI:** 10.1038/s41598-020-60672-5

**Published:** 2020-03-02

**Authors:** Kristen M. Seiler, Adam Bajinting, David M. Alvarado, Mahama A. Traore, Michael M. Binkley, William H. Goo, Wyatt E. Lanik, Jocelyn Ou, Usama Ismail, Micah Iticovici, Cristi R. King, Kelli L. VanDussen, Elzbieta A. Swietlicki, Vered Gazit, Jun Guo, Cliff J. Luke, Thaddeus Stappenbeck, Matthew A. Ciorba, Steven C. George, J. Mark Meacham, Deborah C. Rubin, Misty Good, Brad W. Warner

**Affiliations:** 1 Division of Pediatric Surgery, Department of Surgery, Washington University School of Medicine, St. Louis, Missouri United States; 2 Saint Louis University School of Medicine, St. Louis, Missouri United States; 3 Division of Gastroenterology and the Inflammatory Bowel Diseases Center, Department of Internal Medicine, Washington University School of Medicine, St. Louis, Missouri United States; 4 Department of Biomedical Engineering, Washington University, St. Louis, Missouri United States; 5 Department of Mechanical Engineering & Materials Science, Washington University McKelvey School of Engineering, St. Louis, MO United States; 6 Washington University, St. Louis, Missouri United States; 7 Division of Newborn Medicine, Department of Pediatrics, Washington University School of Medicine, St. Louis, Missouri United States; 8 Division of Pediatric Gastroenterology, Hepatology, and Nutrition, Department of Pediatrics, Cincinnati Children’s Hospital Medical Center and the University of Cincinnati College of Medicine, Cincinnati, OH United States; 9 Department of Pathology & Immunology, Washington University School of Medicine, St. Louis, Missouri United States; 10 Department of Biomedical Engineering, University of California, Davis, California United States

**Keywords:** Gastrointestinal models, Tissue engineering

## Abstract

The development and physiologic role of small intestine (SI) vasculature is poorly studied. This is partly due to a lack of targetable, organ-specific markers for *in vivo* studies of two critical tissue components: endothelium and stroma. This challenge is exacerbated by limitations of traditional cell culture techniques, which fail to recapitulate mechanobiologic stimuli known to affect vessel development. Here, we construct and characterize a 3D *in vitro* microfluidic model that supports the growth of patient-derived intestinal subepithelial myofibroblasts (ISEMFs) and endothelial cells (ECs) into perfused capillary networks. We report how ISEMF and EC-derived vasculature responds to physiologic parameters such as oxygen tension, cell density, growth factors, and pharmacotherapy with an antineoplastic agent (Erlotinib). Finally, we demonstrate effects of ISEMF and EC co-culture on patient-derived human intestinal epithelial cells (HIECs), and incorporate perfused vasculature into a gut-on-a-chip (GOC) model that includes HIECs. Overall, we demonstrate that ISEMFs possess angiogenic properties as evidenced by their ability to reliably, reproducibly, and quantifiably facilitate development of perfused vasculature in a microfluidic system. We furthermore demonstrate the feasibility of including perfused vasculature, including ISEMFs, as critical components of a novel, patient-derived, GOC system with translational relevance as a platform for precision and personalized medicine research.

## Introduction

Blood vessels supply oxygen and nutrients needed to sustain life. As such, their structure and response to physiological stimuli are crucial to nearly all aspects of human biology. This includes normal developmental and pathological conditions affecting the SI such as short gut syndrome, inflammatory bowel disease, and cancer^1–6^. Despite its critical importance, SI vasculature remains poorly studied: One of the reasons for this is inadequacy of *ex vivo* culture systems. Here, we aim to address this void in gastrointestinal (GI) research by applying a microfluidic approach.

Microfluidic technologies offer the unique opportunity for more sophisticated *ex vivo* studies of blood vessel development as compared to static tube formation or proliferation assays as examples. Of importance to GI research, GOC microfluidic platforms capture critical mechanobiological parameters, most notably, fluid flow and interstitial pressure. In 2013, the *ex vivo* development of perfused capillary networks using microfluidic cell culture was first reported by Moya *et al*.^7^. The perfusability of vasculature in this system constituted a crucial development in blood vessel culture, as it is implicitly more physiologically relevant as compared to standard, non-perfused assays.

Like other *ex vivo* models that isolate tissues from the complex *in vivo* environment, microfluidic cell culture allows uncoupling of complex mechanisms into specific hypothesized pathways of interest^8^. For example, ECs require a stromal support cell for efficient development of vessels containing perfused lumens^9^, and so the microfluidic platform allows interrogation of interactions between ECs and other tissue types during vessel formation. The referenced Moya *et al*. study involved co-culture of commercially available normal human lung fibroblasts and ECs embedded in fibrin gels adjacent to microfluidic media lines^7^. To date, other stromal populations such as mesenchymal stem cells and cancer-associated fibroblasts have been used in this system to support perfused capillary network formation via secreted and/or mechanical stimuli on ECs^7,10,11^.

The aim of this study was to develop an *ex vivo* model of SI angiogenesis, to demonstrate the relevance of angiogenesis to epithelial development/function, and to incorporate this into a novel GOC system. In doing so, our goal was to create a physiologically relevant SI culture system that more accurately reflects SI tissue structure as compared to other GOC systems. To enhance the clinical translatability of our system, we sought to use entirely patient-derived tissues. To this end, we incorporate a novel stromal cell type—the ISEMF—and characterize its angiogenic properties. ISEMFs are critical components of the epithelial intestinal stem cell niche, and also affect intestinal immune function^12–15^. We hypothesize that ISEMFs are multifunctional cells that act not only in these described capacities, but also orchestrate angiogenic responses within the mucosa. Thus, we focus significant effort on characterizing vessel formation in response to various stimuli and culture conditions. We also demonstrate a potent effect of angiogenic ISEMF and EC interactions on HIECs, laying a foundation for future study.

In summary, here we leverage microfluidic GOC technology and utilize patient-derived ISEMFs and ECs to develop a platform with which to study SI angiogenesis *ex vivo*. We demonstrate the usefulness of this culture system by interrogating the effects of varying physiologic stimuli and pharmacotherapy on SI angiogenesis, and we demonstrate a potent effect of angiogenesis on HIECs. Finally, we incorporate this perfused capillary system into a patient-derived GOC platform for use in future translational study.

## Results

### Co-culture of patient-derived intestinal subepithelial myofibroblasts and endothelial cells in microfluidic devices generates perfused, visually quantifiable, capillary networks

**Figure 1:**
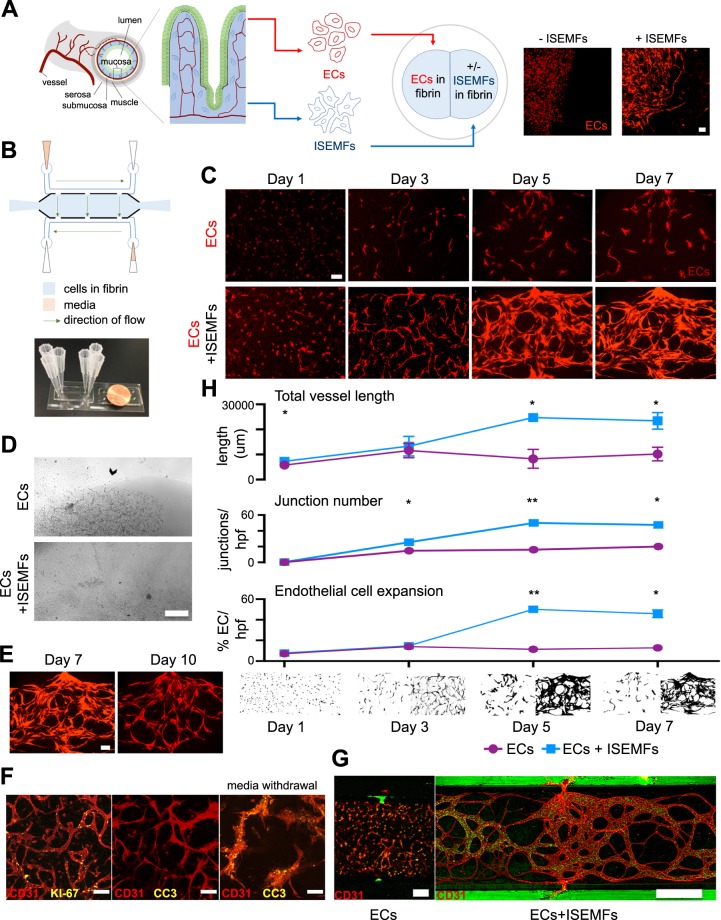
Co-culture of patient-derived ISEMFs and ECs in microfluidic devices to generate perfused vasculature. (**A**) Schematic representation of SI mucosa, showing its capillary network, to be modeled *ex vivo* using ECs and ISEMFs. Representative images of fluorescent ECs cultured in the presence (right) or absence (left) of ISEMFs after 24 h are shown. Magnification: 10×, scale bar: 100 μm. (**B**) Schematic representation of microfluidic device, with actual device beside a penny for size comparison. The central culture chamber abuts media lines, synapsing with them via pores, resulting in a net flow of media and interstitial pressure across the chamber. (**C**) Effect of EC monoculture (above) vs. co-culture with ISEMFs (below) on vessel development over 7 days (n = 2 per condition). Magnification: 10×, scale bar: 100 μm. (**D**) Images of standard well-plate culture (as in **1A**) of ECs cultured beside fibrin (above), vs ECs cultured beside fibrin-suspended ISEMFs (below), after 7 days in culture. Arrow highlights stability of adjacent fibrin in the absence of ISEMFs vs digestion and collapse of the culture system in the presence of ISEMFs. Magnification 4×, scale bar: 500 μm. This is compared to microfluidic culture (**E**), where vessel involution in not seen until day 10. Magnification 10×, scale bar: 100 μm. (**F**) Immunofluorescence co-localization of EC marker CD31 with proliferation marker KI-67 (left), and cell death marker CC3 (middle/right), on day 3 of culture. CC3 expression was only appreciable after media withdrawal (right). Magnification: 10×, scale bar: 100 μm. (**G**) Microfluidic culture of ECs alone (left, magnification 10×, scale bar: 100 μm), as compared to ECs + ISEMFs (right, magnification 4×, scale bar: 500 μm). 10 μm fluorescent beads (green) flowed through capillaries, which synapsed with microfluidic lines at the pores, in the EC + ISEMF condition only. (**H**) Quantification of angiogenesis in the presence (blue) or absence (purple) of ISEMFs, as in **1C**. Measurements (from top to bottom) included total vessel length, junction number per hpf, and EC expansion (percent fluorescent ECs per hpf, on binary image analysis). Representative binary images are shown below graphs, with ECs (left) and ECs+ ISEMFs (right) at each time point. Graphs are mean +/− SD. *P < 0.05, **P < 0.01.

Historically, fibroblasts have been used to elicit angiogenesis/vasculogenesis *in vitro*, and so we first aimed to determine whether patient-derived ISEMFs—which have features of both fibroblasts and myocytes^16^— influence angiogenic activity of ECs^9,17^. We initially used a simple co-culture technique as shown in Fig. 1A. ECs were transfected to express fluorescent protein, which allows visualization of vessel development over time. These ECs were suspended in fibrin gel and cultured in a standard well-plate immediately adjacent to either fibrin gel, or fibrin gel containing patient-derived ISEMFs (obtained from surgical samples at Washington University). When cultured beside plain fibrin gels, ECs demonstrated minimal elongation, organization and branching activity (Fig. 1A). In contrast, ECs cultured in the presence of ISEMFs organized into elongated, vessel-like structures which invaded the adjacent fibrin gel within 24 h (Fig. 1A). This suggested patient-derived ISEMFs are indeed capable of supporting vasculogenesis, similar to fibroblast cell types that have been explored in similar systems^7,10,11^.

Given our finding that ISEMFs induce EC formation of vessel-like structures, we next sought to evaluate how to incorporate perfused vasculature into GOC. To create a translational system of ISEMF-induced angiogenesis, we fabricated microfluidic devices of the design indicated in Fig. 1B. The tissue culture chamber connected via pores with microfluidic lines carrying media, which flowed directionally through the media lines as shown (Fig. 1B). We expected vessels cultured in this system would synapse with these pores, similar to previously published findings^7^. Further, because one media line was supplied with more media than the other, a subtle intermittent hydrostatic pressure across the central tissue culture chamber (from high media reservoir to low) was created, as reported earlier^18^. Interstitial pressure is an important mechanobiologic stimulus experienced by vasculature *in vivo*, and microfluidic culture captures this *ex vivo* in ways static culture cannot.

Co-culture of fibrin-embedded ISEMFs and ECs in this microfluidic system elicited the spontaneous development of capillary networks which synapsed with pores connecting the central tissue culture chamber to the media lines (Fig. 1C). To demonstrate that mechanobiologic stimuli of fluid flow and interstitial pressure were insufficient to induce capillary development of ECs alone, we performed a time course experiment of EC mono-culture vs EC + ISEMF co-culture, showing that ECs alone did not form capillary networks, while the addition of ISEMFs induced capillary network formation within 3–5 days (Fig. 1C). Vessel networks reliably persisted until 7 days in culture. Interestingly, we observed rapid disintegration of fibrin gels and vessel like structures beginning within 3 days in well-plate assays, with complete collapse by day 7 (Fig. 1D). This suggested our co-cultured cells were mechanosensitive, and that intermittent hydrostatic pressure dampens aggressive, matrix metalloproteinase and secreted factor mediated stromal cell behavior, which is known to occur in response to angiogenic stimuli^9,19,20^. In the microfluidic devices, vessel involution was reliably appreciable by day 10 (Fig. 1E).

To localize proliferative and/or apoptotic ECs during vessel development, we performed immunofluorescence co-localization studies of EC marker CD31, proliferation marker KI-67, and apoptosis marker cleaved caspase 3 (CC3) on day 3 of device culture. As shown in Fig. 1F, proliferative ECs are found throughout vessel structures, and there is no evidence of apoptotic ECs. This is consistent with the angiogenic process, wherein stalk cells proliferate to achieve elongation/lumen formation, and are guided and ‘pulled’ by the migrating tip cell^21–23^. To confirm CC3 antibody reactivity, we withdrew culture media for 3 hours and then repeated staining to identify apoptotic cells (Fig. 1F).

Because perfusion is a critical physiologic function of vessels, we wanted to conclusively demonstrate perfusion and structural integrity of our microfluidic capillary networks. We did so by introducing 10 μm fluorescently labeled beads through the media lines (Fig. 1G). Fluorescent beads entered and exited vessel lumens through synapsing pores between the media lines and central culture chamber, but did not filter through vessel walls, demonstrating the presence of intact, hollow, capillary structures. Vessels with perfusable lumens did not develop in the absence of ISEMFs (Fig. 1G), again reiterating the importance of ISEMFs to EC behavior.

A primary advantage of microfluidic cell culture for studying SI angiogenesis is the ability to obtain visual analyses of 3D vascular structures. For example, the height of the microfluidic tissue culture chamber was ~100 μm, allowing consistent, easy visualization of cell activity and vessel formation/structure. On the other hand, we had difficulty visually isolating structures of interest in well-plate assays, owing to multiple, overlapping, fluorescent vessels in 3D gels which were prone to rapid collapse (as in Fig. 1A,D). This made analysis of angiogenic activity cumbersome, underscoring another substantial advantage of microfluidic as compared to static culture.

Given the optical transparency of microfluidic devices, we next sought to quantify vessel development in ISEMF and EC co-culture. To do so, we employed both manual (using ImageJ) and automated (vessel mapping using AngioTool^24^) methods. First, we quantified total vessel length (the sum of all vessel lengths per high powered field, or hpf), in the presence or absence of ISEMFs (Fig. 1H). This revealed a significant increase in total vessel length in the presence of ISEMFs at days 1 (5759 ± 15.6 μm without ISEMFs, vs 7260 ± 1972 μm with ISEMFs, *P* < 0.05), 5 (8290 ± 2655 μm without ISEMFs, vs 24705 ± 519.9 μm with ISEMFs, *P* < 0.05), and 7 (10175 ± 1912 μm without ISEMFs, vs 23431 ± 2332 μm with ISEMFs, *P* < 0.05) (Fig. 1H). Second, we measured the total number of junctions (a measure of vessel branching/network complexity) per hpf, which demonstrated a significant increase in the presence of ISEMFs at days 3 (14.7 ± 2 junctions without ISEMFs, vs 25.5 ± 0.8 junctions with ISEMFs, *P* < 0.05), 5 (16 ± 3 junctions without ISEMFs, vs 50 ± 1 junctions with ISEMFs, *P* < 0.01), and 7 (20 ± 2 junctions without ISEMFs, vs 47.5 ± 2 junctions with ISEMFs, *P* < 0.05) (Fig. 1H). Finally, we measured EC expansion (a surrogate for EC proliferation over time, using binary image analysis of fluorescent ECs), revealing a significant increase in the presence of ISEMFs at days 5 (11.5 ± 0.9% fluorescent ECs per hpf without ISEMFs, vs 50.1 ± 1.3% with ISEMFs, *P* < 0.01) and 7 (12.8 ± 2.4% fluorescence per hpf without ISEMFs, vs 45.8 ± 27% with ISEMFs, *P* < 0.05) (Fig. 1H).

Taken together, these results demonstrate a clear effect of patient-derived ISEMFs on ECs, eliciting angiogenesis when co-cultured. Microfluidic co-culture of these cells induces spontaneous development of perfused capillary networks, and structural features of these networks are readily quantifiable.

### Co-culture of ISEMFs with ECs induces changes in ISEMF stromal marker gene expression, while EC identity is unperturbed

Considering fibroblasts and vascular pericytes are the stromal lineages most commonly implicated in angiogenesis, we wanted to determine whether ISEMFs shift identity in response to angiogenic stimuli to become more like either of these lineages. To determine this, we cultured ISEMFs in a basal medium (which is used to isolate and expand the cells, and contains no exogenous growth factors), and compared gene expression of ISEMFs under this condition to two others conditions: First, we tested the effect of angiogenic growth medium (EGM-2 media). Second, we used a standard Transwell assay to determine whether there was an additive effect of ECs on ISEMFs exposed to angiogenic medium. For each condition, we isolated mRNA from ISEMFs and performed RT-qPCR for stromal cell markers vimentin (*VIM*, enriched in fibroblasts and myofibroblasts), desmin (*DES*, enriched in vascular pericytes), and α-smooth muscle actin (α-SMA, or *ACTA2*, enriched in ISEMFs and vascular pericytes)^25^.

**Figure 2:**
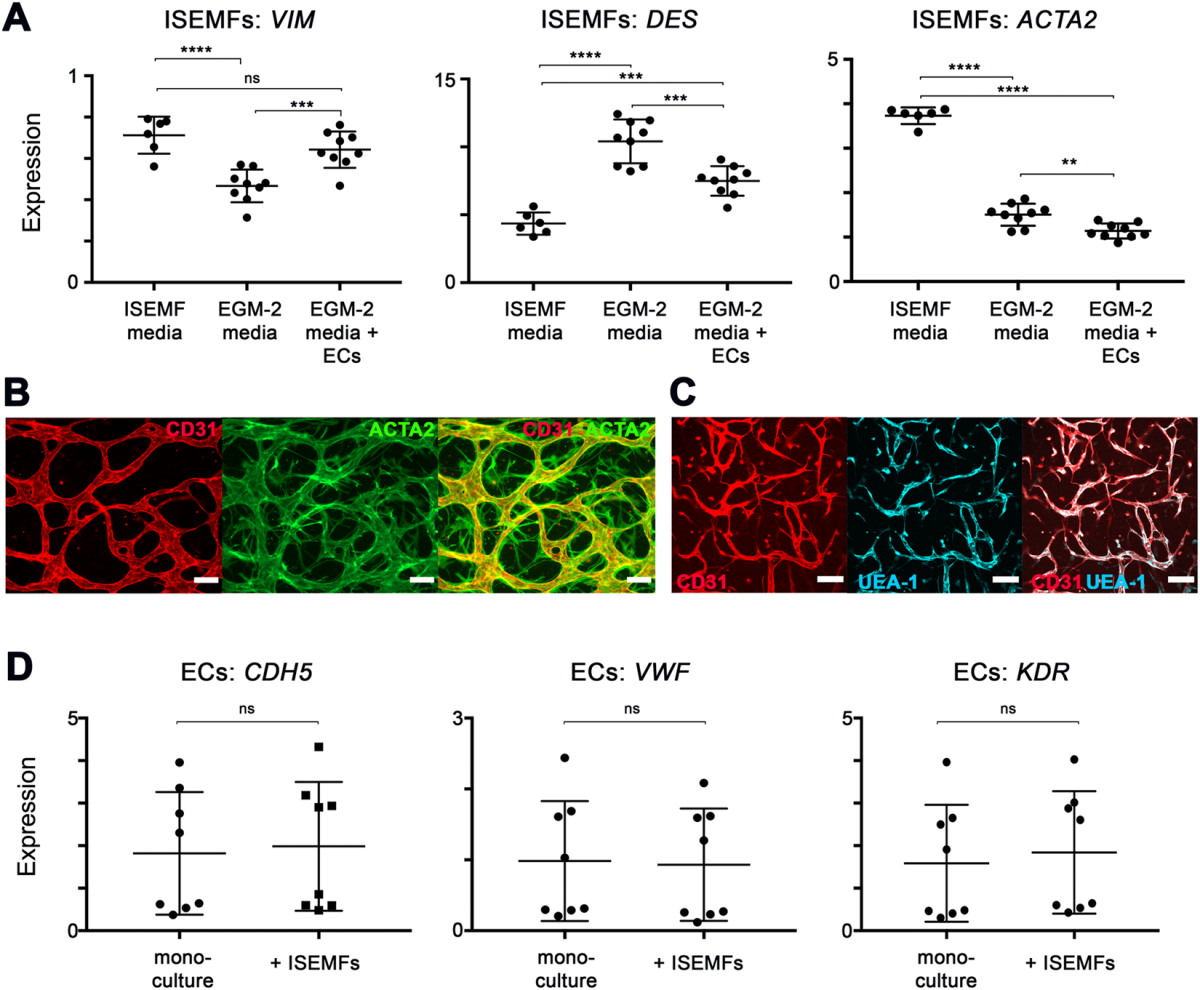
Assessing the identity of ISEMFs and ECs in response to one another. (**A**) Relative expression of stromal lineage markers *VIM*, *DES*, and *ACTA2* in ISEMFs grown in the presence of native media (ISEMF media, n = 6) vs. angiogenic media (EGM-2 media, n = 9), and also in the presence of EGM-2 media with co-cultured ECs in a Transwell system (n = 9). Co-cultures were performed overnight. (**B**) Immunofluorescence staining images of CD31 (ECs) and *ACTA2* (ISEMFs), demonstrating their structural relationship (taken on day 5 of culture). Magnification: 10×, scale bar: 100 μm. (**C**) Immunofluorescence co-localization staining images of EC markers CD31 and UEA-1 (taken on day 3 of culture). Magnification: 10×, scale bar: 100 μm. (**D**) Relative expression of EC markers *CDH5*, *VWF*, and *KDR* in ECs in mono-culture (n = 8) vs co-culture with ISEMFs in a Transwell system (n = 8). Co-cultures were performed overnight. **P < 0.01, ***P < 0.001, ****P < 0.0001, ns = not significant.

As shown in in Fig. 2A, exposure of ISEMFs to angiogenic conditions induced a 65.6% reduction in *VIM* expression (*P* < 0.0001), a 239.1% increase in *DES* expression (*P* < 0.0001), and a 40.3% decrease in *ACTA2* expression (*P* < 0.0001). Interestingly, the addition of ECs reversed the effect of EGM-2 media on *VIM* expression, partially mitigated the effect of EGM-2 media on *DES* expression, and had an additive effect with EGM-2 media on the suppression of *ACTA2* expression (*P* < 0.01). That said, ISEMFs do continue to express *ACTA2* in device culture, forming a sheath-like structure around vessels formed by CD31+ ECs (Fig. 2B). Overall, these results indicated to us that ISEMF identity and phenotype are altered by angiogenic stimuli, and further altered by the presence of ECs, suggesting an instructive “cross-talk” between these cells. Further work is warranted to more precisely characterize the identity and behavior of ISEMFs during angiogenesis, as our results suggest a “hybrid” expression of multiple stromal markers.

Recognizing that ECs affect ISEMF gene expression, we also wanted to determine whether EC identity is maintained in the presence of ISEMFs. We thus performed two experiments. First, we verified EC functional identity during co-culture in devices by demonstrating co-expression of EC-specific protein Ulex europaeus agglutinin-1 (UEA-1) with CD31+ (Fig. 2C). Second, we performed Transwell assays similar to above, and measured the mRNA expression of typical EC markers vascular endothelial cadherin (also known as cadherin 5, or *CDH5*), von Willebrand factor (*VWF*), and vascular endothelial growth factor receptor 2 (also known as kinase insert domain receptor, or *KDR*). As shown in Fig. 2D, the addition of ISEMFs had no effect on the expression of these EC markers. This suggests ECs maintain their identity when cultured in the presence of ISEMFs.

### ISEMF and EC-derived vasculature is quantitatively responsive to physiologic stimuli including oxygen tension, cell concentration, growth factors, and pharmacotherapy

Given our findings thus far, we next sought to demonstrate the translational relevance of our system by characterizing the responsiveness of the capillaries to various physiologic stimuli. These included oxygen tension, cell density, growth factor exposure, and pharmacotherapy.

**Figure 3:**
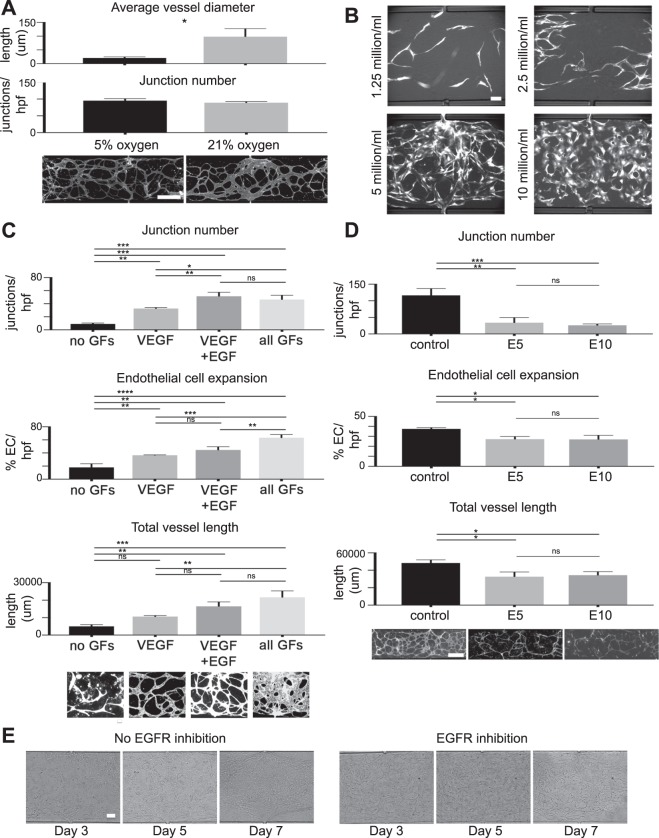
Measuring the responsiveness of ISEMF and EC-derived vasculature to physiologic stimuli. (**A**) Beginning at time of device loading, microfluidic devices were cultured in either 5% (left) or 21% (right) oxygen (n = 3 per condition). Measured endpoints were vessel diameters (above) and number of junctions (a measure of vessel branching/network complexity) per high powered field (hpf, below). Representative images of vessels at 5% and 21% oxygen are shown. Magnification: 4×, scale bar: 500 μm. (**B**) Representative images of vessels using loading cell densities of 1.25, 2.5, 5, and 10 million ECs per ml of fibrin, with a 1:3 ratio of ISEMFs to ECs, after 7 days in culture. Magnification: 10×, scale bar: 100 μm (**C**). Beginning at the time of device loading, cells were cultured in the following conditions (from left to right): no growth factors (n = 2), VEGF only added (n = 3), VEGF and EGF added (n = 3), and all growth factors from EGM-2 media (n = 3). Measured endpoints were (from top to bottom): junction number per hpf, endothelial cell expansion (as determined on binary image analysis), and total vessel length (i.e. the sum of all vessel lengths per hpf). Representative images of vessels cultured under each of these conditions are shown. Magnification: 10×, scale bar: 100 μm. (**D**) Beginning at the time of device loading, cells were cultured with vehicle control (EGM-2 media +DMSO), or in the presence of EGFR inhibitor Erlotinib at a concentration of 5 or 10 μM (n = 3 for each condition). Measured endpoints from top to bottom: junction number per hpf, endothelial cell expansion (as determined on binary image analysis), and total vessel length per hpf. Representative images of vessels cultured under each of these conditions are shown. Magnification: 4×, scale bar: 500 μm. (**E**) The effect of EGFR inhibition on established vessel networks was tested by adding Erlotinib (10 μM) after 3 days of standard culture (right), vs continuing standard culture conditions (left). Representative bright field images are shown. Magnification: 10×, scale bar: 100 μm. All graphs are presented as mean +/− SD. *P < 0.05, **P < 0.01, ***P < 0.001, ****P < 0.0001, ns = not significant.

First, we examined the effect of oxygen concentration on vessel network structure. Because capillaries *in vivo* typically experience ~5% oxygen^26,27^, we hypothesized 5% oxygen tension would induce formation of thinner, more highly branched vessels as compared to higher/atmospheric oxygen tension (~21%), which is typically experienced by larger arteries *in vivo*. Moreover, we hypothesized that oxygen is a biologically relevant stimuli that affects vessel structure, and that its effect could be quantified in our devices. To test this, we performed culture in either a 5 or 21% oxygen environment, and quantified average vessel diameter. This revealed significant increases in vessel diameter in 21% as compared to 5% oxygen (98 ± 16.9 μm average vessel diameter in 21% oxygen, vs 20.5 ± 2.4 μm in 5% oxygen, *P* < 0.05) (Fig. 3A). This result is notable because cell culture is commonly performed at atmospheric oxygen, which may not be the most physiologically relevant environment, especially for studies of GI mucosal biology wherein the physiologic oxygen tension is less than 10%^27^. We also observed a small but insignificant increase in number of junctions per hpf in 5% as compared to 21% oxygen (95.5 ± 3.4 junctions per hpf at 5%, vs 89 ± 2.1 at 21%, *P* = 0.19).

Second, because cell culture density is known to have a substantial effect on experimental outcome owing to paracrine signaling^28^, we tested the effect of altering cell concentrations of ISEMFs and ECs. We investigated four different loading cell densities, each sequential density being double to the one prior (Fig. 3B). Of note, ISEMFs are large cells relative to ECs, and while the final ISEMF to EC ratio was approximately 1:1 with regard to pelleted cell volume, the numerical ratio was approximately 1:3. Using higher ratios of ISEMFs to ECs was associated with rapid fibrin degradation and vessel collapse (not shown). As such, reported densities (Fig. 3B) are relative to ECs, with final EC concentrations of 1.25, 2.5, 5, or 10 million cells/ml of fibrin. We found that the highest cell density (10 million ECs/ml) tended to yield obscured, enlarged vessel morphology (Fig. 3B). This was undesired, as it made quantitation using image analysis software (AngioTool, which is easier to calibrate to thinner vessels) more tedious. The lowest cell density (1.25 million ECs/ml) did not generate perfused vessels (Fig. 3B). We had variable success generating vessels at 2.5 million ECs/ml, and consistent success with 5 million ECs/ml (Fig. 3B). Given these findings, we conclude that cell density and cell ratios are physiologically relevant factors that needs to be titrated and kept consistent across experimental conditions.

Third, we tested the additive effect of growth factors (GFs) from EGM-2 media on vessel development. The EGM-2 bullet kit contains GF supplements consisting of insulin-like growth factor (IGF), vascular endothelial growth factor (VEGF), epidermal growth factor (EGF), and fibroblast growth factor (FGF), which are individually added to the endothelial basal media (EBM) at proprietary concentrations. Characterizing the additive effects of these GFs was important because the strong pro-angiogenic stimuli of completely reconstituted EGM-2 may mask effects of additional permutations, similar to how the originally published SI organoid culture conditions mask the additive effects of novel biological stimuli on organoid proliferation^29–32^. Furthermore, GFs that induce angiogenesis may have undesired off-target effects on HIECs in a combined GOC system.

Therefore, to test the additive effect of EGM-2 GFs on ISEMF-induced angiogenesis, we first removed all four GFs (Fig. 3C). Under this specific condition, vessels did not form, suggesting microfluidic culture alone is insufficient to induce perfused capillary formation in the presence of ISEMFs, and additional growth factor stimulus is needed.

Because VEGF is a prototypical and well-studied pro-angiogenic molecule^33,34^, we next tested the effect of adding only VEGF to the system. We found that VEGF alone was sufficient to induce vessel formation (Fig. 3C), though the response was less robust as compared to a positive control containing all four GFs. Results were as follows: 32.7 ± 0.9 junctions per hpf with VEGF only, vs. 46.3 ± 3.8 with all four GFs, *P* < 0.001; 36.4 ± 0.4% fluorescent ECs per hpf with VEGF only, vs. 63.2 ± 2.9% with all four GFs, *P* < 0.0001; 10592 ± 345.3 μm total vessel length per hpf with VEGF only, vs. 21639 ± 2133 μm with all four GFs, *P* < 0.001 (Fig. 3C). We concluded that adding only VEGF to EBM induces angiogenesis, while also allowing the addition of synergistic factors to achieve a desired effect which may otherwise be masked. Furthermore, for future GOC studies, this method can ensure capillary development occurs, while minimizing off-target epithelial effects.

For example, EGF has well described effects on the intestinal epithelium, including intestinal adaptation^35–37^. Further, epidermal growth factor receptor (EGFR) signaling is important during adaptive angiogenesis^1,38^. This said, we were interested in a potential synergistic effect of EGF and VEGF on vessel formation and structure in our ISEMF-EC model. Indeed, we did observe an increase in all quantitative endpoints when EGF was added with VEGF, as compared to VEGF only, though our results only reached significance with regard to junction number (Fig. 3C). VEGF + EGF results (and the *P*value when compared to VEGF only result, presented above) were as follows: 51.3 ± 3.5 junctions per hpf, *P* < 0.01; 44.5 ± 2.9% fluorescent ECs per hpf, *P* = 0.19; 16463 ± 1466 μm total vessel length per hpf, *P* = 0.08. These results suggest EGFR signaling affects ISEMF-mediated vasculogenesis, particularly with regard to vessel branching/structural remodeling, and deserves further study.

Finally, given these observations we next wanted to test the responsiveness of ISEMF and EC-derived vasculature to an EGFR inhibitor. Erlotinib is an EGFR tyrosine kinase inhibitor with speculated but poorly characterized anti-angiogenic properties^39–41^: Erlotinib is used clinically as a synergistic anti-neoplastic agent, lending credence to the importance of testing its efficacy in a personalized manner, *ex vivo*. We performed our study using two concentrations of Erlotinib: 5 μM and 10 μM. These concentrations were selected based on prior *in vitro* studies of Erlotinib pharmacology^41–43^, as well as data on long-term trough levels of Erlotinib in pancreatic cancer patients^44^.

We found that compared to vehicle control, Erlotinib blunted vessel network formation at both 5 μM and 10 μM concentrations, with no significant difference in results between 5 and 10 μM dosing (Fig. 3D). There were 116.3 ± 11.8 junctions per hpf with vehicle control, vs. 34 ± 9.0 with 5 μM Erlotinib, *P* < 0.01; 37.5 ± 08% fluorescent ECs per hpf with vehicle control, vs. 27.2 ± 1.5% with 5uM erlotinib, *P* < 0.05; 48343 ± 2080 μm total vessel length per hpf with vehicle control, vs. 32591 ± 3096 μm with 5 μM erlotinib, *P* < 0.05 (Fig. 3D). In this experiment, Erlotinib was added on day 0 (time of microfluidic device loading), before vessel networks were established. As such, we also wanted to determine the effect of EGFR inhibition on established vessel networks, as this likely has clinical relevance, potentially affecting the side effect profile of the drug (predominantly rash and diarrhea)^45^. We found that adding Erlotinib to established vasculature had the effect of preventing vessel remodeling which otherwise occurs over time in culture (Fig. 3E). These results indicated that Erlotinib has a major effect on angiogenesis, and that this effect is intrinsic to the drug itself rather than purely synergistic in combination with other antineoplastic agents, as previously reported^39–41^.

Collectively, we have developed a system in which patient-derived ISEMFs and ECs develop into perfused capillaries that can be experimentally manipulated to quantitatively test the effects of relevant physiologic stimuli including oxygen tension, paracrine signaling (cell density), growth factors, and a commercially available pharmaceutical agent. The latter, in particular, highlights the translational relevance of this system with regard to personalized or precision medicine.

### Integration of ISEMF and EC perfused vasculature into a Gut-on-a-Chip model that includes patient-derived intestinal epithelium

It has been previously demonstrated that endothelial cells affect intestinal epithelial proliferation and barrier function^46^. This study was performed by culturing a monolayer of epithelium above a monolayer of endothelium^46^. Similarly, other studies have demonstrated an effect of ISEMFs on epithelial development and function^14,47^. We were thus curious to determine whether there was an additive effect of ECs and ISEMFs on epithelial biology. If so, this would encourage future work to develop a system that incorporates perfused vasculature below a monolayer of patient-derived HIECs, so as to model “systemic circulation” and “luminal flow” simultaneously *ex vivo*. This is a logical next step toward personalized medicine in GI research.

**Figure 4:**
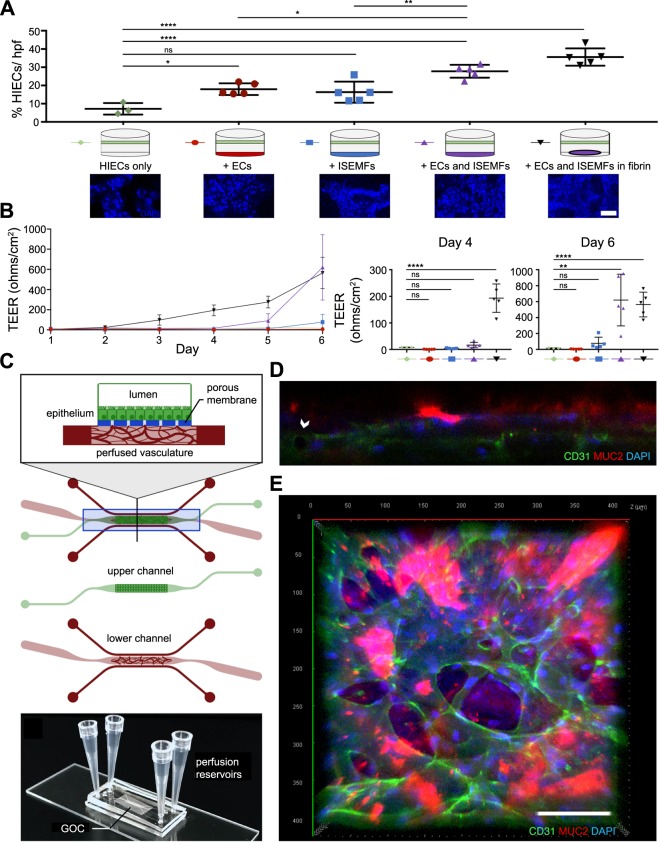
Effects of ECs and ISEMFs on the expansion and TEER of patient-derived HIECs, and integration of perfused vasculature into a patient-derived GOC model that includes HIECs. (**A**) Measurement of HIEC expansion (as determined on binary image analysis of DAPI staining) when grown on Transwell inserts above conditions as shown (n = 3 for HIEC mono-culture, n = 5 for all other conditions). Representative images of DAPI stained HIECs are shown. Magnification: 40×, scale bar: 50 μm. (**B**) Measurement of HIEC TEER, assessed daily over the course of 6 days, when grown on Transwell inserts above conditions as shown (n = 3 for HIEC mono-culture, n = 5 for all other conditions). Statistical differences between conditions on days 4 and 6 of culture are presented in the panels to the right. (**C**) Illustration of combined vasculature and epithelium GOC model. Tissues are cultured in a channel-over-channel design, separated by a thin microporous membrane, with perfused vasculature in the lower channel, and ileal HIECs as a monolayer (atop the membrane) in the upper channel. The lower channel is perfused by adjacent microfluidic media lines, allowing isolation of luminal flow (upper chamber) and systemic circulation (perfused vasculature). (**D**) Confocal Z-stack image shows epithelium above vasculature. Ileal epithelium has apical secretion of mucin (MUC2, red), and DAPI nuclear counterstain (blue) indicates basolateral nuclear localization, indicative of epithelial polarity when cultured as a monolayer in the upper chamber, with vasculature (indicated by CD31, green) in the lower chamber. Arrow indicates vessel lumen. (**E**) Top down view of GOC, with staining as in 4D. Magnification: 20×, scale bar: 100 μm. All graphs are presented as mean +/− SD. *P < 0.05, **P < 0.01, ****P < 0.0001, ns = not significant.

To test the additive effects of ECs and ISEMFs on HIEC biology, we performed Transwell assays as shown in Fig. 4A. Briefly, HIECs (obtained from ileal biopsy samples at Washington University) were cultured on Transwell inserts either alone, above ECs, above ISEMFs, above ECs and ISEMFs cultured together on the well plate directly, or above ECs and ISEMFs suspended together in fibrin. The experiment was carried out over 6 days, with transepithelial electrical resistance (TEER) measurements performed daily to assess permeability of the HIEC monolayer. At the conclusion of the experiment, relative expansion/confluence of HIECs was assessed via quantification of nuclear staining. As shown in Fig. 4A, co-culture with ECs more than doubled HIEC confluence relative to HIEC mono-culture (2.49-fold increase, *P* < 0.05). Co-culture with ISEMFs had a similar effect (2.26-fold increase in HIEC confluence), however this result did not reach significance (*P* = 0.06). Notably, the effect on HIECs was enhanced by EC and ISEMF co-culture (3.85-fold increase, *P* < 0.0001), and even further amplified when ECs and ISEMFs were co-cultured together in fibrin (4.9-fold increase, *P* < 0.0001).

We observed a similar effect on TEER measurements of the HIEC monolayer. Here, co-culture of ECs and ISEMFs in fibrin led to an early and striking increase in HIEC TEER when compared to mono-culture (35.4-fold increased TEER at day 6, *P* < 0.01) or co-culture with ECs alone or ISEMFs alone (0.36-fold decrease, *P* > 0.99 and 4.8-fold increase,* P* = 0.99, respectively) (Fig. 4B). Co-culture of ECs and ISEMFs without fibrin also had a significant enhancing effect on HIEC TEER, however, this effect was delayed until later in culture (day 6). At the same time, the additive effect of fibrin to co-culture of ECs with ISEMFs waned by day 6, possibly owing to digestion and collapse of fibrin gels by this point (Fig. 1D).

Collectively, these results suggest that the interaction of ISEMFs and ECs has a potent effect on HIECs proliferation and function as assessed by TEER, more so than the effect of ECs or ISEMFs alone. Further, suspension of these two cell types in a fibrin matrix—an environment in which matrix remodeling and angiogenesis can occur—directly and dramatically affects SI epithelial physiology. This is a novel finding, and one which merits further investigation.

As such, to construct a more complex and translational model of GOC, we modified our microfluidic device design as shown in Fig. 4C. Essentially, we constructed a channel-over-channel device, with the channels separated by a microporous membrane, similar to previously published work^46,48,49^. However, instead of a single-chamber culture channel on the bottom, we incorporated adjacent microfluidic media lines (Fig. 4C). Additionally, instead of a polydimethylsiloxane (PDMS) membrane, we incorporated a collagen-IV treated polycarbonate membrane. The lower channel was used to culture vasculature, as described above, and patient-derived HIECs were cultured on the apical side of the membrane in the upper channel, as a monolayer (Fig. 4C–E). In this system, polarized HIEC growth was demonstrated by apical mucin (MUC2) secretion from differentiated goblet cells (Fig. 4D,E).

In summary, here we demonstrate that SI perfused vasculature can be co-cultured in the bottom, “subepithelial” channel of a GOC platform, establishing our model as a “proof of principle” for the co-culture of multiple patient-derived intestinal tissues in a perfused, microfluidic model of intestinal physiology.

## Discussion

The overarching goal in this study was to develop an *ex vivo* model of SI angiogenesis, and to incorporate ISEMFs and perfused vasculature into a more physiologically relevant GOC system. ISEMFs have multiple important roles in GI mucosal physiology^13,14,50^. As such, the relevance of their inclusion in *ex vivo* GI culture systems is inherent.

To our knowledge, this is the first report wherein patient-derived ISEMFs have been used to create perfused capillary networks *ex vivo*, including its subsequent integration into GOC. This opens avenues to studying SI angiogenesis in a patient and disease-specific manner. Furthermore, this novel culture technique allows for more refined experiments of intestinal systemic circulation or leukocyte extravasation—processes that are critically relevant to GI research. It also represents a building block toward more sophisticated GOC technology, wherein both “systemic circulation” and luminal flow could be modeled more elegantly. Our use of patient-derived tissues has translational relevance and implications for precision medicine approaches. A future direction of this study will be to challenge our microfluidic GOC system with conditions mimicking various disease states.

A frequently referenced GOC system has a channel-over-channel design, and the channels are separated by a thin, microporous membrane^51^. This system has been used to demonstrate epithelial monolayer growth on the apical side of the membrane, and endothelial cell monolayer growth on the basal side of the membrane^46^, with isolated “luminal” (apical) and “systemic” (basal) “circulation” (media flow through the channels). It has not incorporated patient-derived ISEMFs, which would be advantageous because they are an important component of the mucosal epithelial niche^12–15^. Nor has it incorporated 3D vasculature, another important component of the mucosal epithelial niche. To incorporate these structural and cellular elements, we contemplated loading cells embedded in fibrin gels into the lower chamber of this standard design. This would be more physiologically relevant not only for the cell types and vasculature included, but also because, *in vivo*, mucosal tissue below the epithelium is solid. However, in the absence of adjacent microfluidic media lines in the lower channel, the system would rely entirely on luminal perfusion. Further, it would be difficult to demonstrate perfusability of the vasculature in this system, as vessel lumens would not be accessible. We felt this lack of perfusability would significantly limit the translational relevance of the system (to investigate vascular transport of nutrients, drugs, and blood components, for example) and so, to create a more translational system of ISEMF-induced vasculogenesis, we fabricated microfluidic devices as in Fig. 4C. Admittedly, our system lacks mechanical peristalsis, which has the advantage of inducing more complex epithelial architecture^48,51^. Moving forward, a GOC which integrates both perfused capillaries and peristalsis (via vacuum^48,51^, spring, or other means) would be worth investigating. Further, regarding long-term culture, we would like to note that vessel structures remain stable in microfluidic devices when FGF and VEGF are removed from the culture media after vessel networks have formed^7^. This is logical, as FGF stimulates aggressive fibroblast activity, resulting in matrix (fibrin) digestion^9,52^.

Considering that organotypic gene expression patterns in ECs have been described^53^, a potential limitation of our model is the use of a progenitor EC population (endothelial colony forming cell-derived endothelial cells, or ECFC-ECs), rather than human intestinal microvascular endothelial cells (HIMECs). Preliminary studies in our lab did demonstrate enhanced angiogenic activity of HIMECs (obtained from collaborator Dr. David Binion, University of Pittsburgh^2^) cultured in the presence of ISEMFs. However, owing to the ease of large-scale expansion of ECFC-ECs, which allows for greater experimental reproducibility, we elected to use this cell population. Certainly, future experiments could focus on specific interactions between ISEMFs and HIMECs, or other EC populations.

There are several principles of ISEMF biology that have been demonstrated by this study, and warrant further investigation. First, we show that patient-derived ISEMFs have angiogenic properties. To our knowledge, this has been speculated or inferred, but not definitively proven. In one report, human umbilical vein endothelial cells (HUVECs) demonstrated increased migration and tube formation when cultured in the presence of PGE-2 stimulated colonic myofibroblasts, but not unstimulated myofibroblasts, suggesting that colonic myofibroblasts mediate inflammatory angiogenesis^54^. Similarly, Schirbel *et al*. showed that human colonic fibroblasts secreted pro-angiogenic factors in response to TLR and NLR stimulation^3^. Moreover, cancer studies have pointed to myofibroblasts as a source of pro-angiogenic factors that fuel tumor angiogenesis^55^. None of these studies have examined the capacity of patient-derived ISEMFs to elicit angiogenesis *ex vivo*, and we hope our findings will facilitate discovery in new areas of GI research.

Second, by demonstrating the selective formation of perfused vessel networks in response to interstitial pressure generated by microfluidic culture—as opposed to standard well-plate culture—we identified a new mechanoresponsive element of patient-derived ISEMFs. A prior report showed that murine intestinal myofibroblasts are mechanoresponsive to extracellular matrix density, which determines organoid contractility^56^. Cellular mechanobiology in the intestine is a highly underdeveloped area of study that warrants further investigation, especially since inflammatory conditions of the SI induce tissue fibrosis (stiffening), and this may in and of itself affect disease susceptibility and progression.

Third, we would also like to highlight that the patient-derived ISEMFs used in this study also support the growth of patient derived intestinal epithelium (Dr. Deborah Rubin, unpublished). This has also been demonstrated in murine studies^14^. Recognizing that ISEMFs affect behavior of multiple cell lineages, and can shift behavior in response to multiple stimuli, we build on an increasing body of literature supporting a critical role for these cells in intestinal biology^13,25,57^. We suggest that ISEMFs act as an “operator” or “switchboard” in the SI, integrating systemic and local environmental cues to direct epithelial, endothelial, and other cellular behaviors simultaneously.

Finally, here we utilized an *ex vivo* model of SI vasculogenesis to quantitatively measure the effects of pharmacologic EGFR inhibition on capillary development. Erlotinib is a receptor tyrosine kinase inhibitor of EGFR, which is approved clinically in certain instances of metastatic non-small cell lung cancer or pancreatic cancer, and its role in suppressing tumor angiogenesis has also been described^40^. However, it was previously unclear whether this is through direct effects on EGFR, through off-target effects, synergistic effects with other antineoplastic agents, or a combination of these. Here, we show that erlotinib inhibits angiogenesis and stabilizes extant vasculature by preventing its remodeling. These findings offer insight into the mechanism of action of this chemotherapeutic agent, and also demonstrate the translatability of our model system as an *ex vivo* culture system for drug testing.

## Materials and Methods

### Microfluidic device microfabrication

Microfluidic molds were fashioned according to standard methods using soft lithography to cast polydimethylsiloxane (PDMS) over SU-8 photoresist molds as previously described^7^. Devices were fabricated using the SYLGARD 184 Silicone Elastomer Kit (NC9285739, Dow Corning; Midland, MI) at a 10:1 PDMS: curing agent ratio, degassed in a vacuum chamber, and then heat cured at 65 °C. For studies of SI vasculogenesis (single chamber design), cured devices were then removed from molds, punched with 16-gauge needles to accommodate pipette tips for media, and plasma bonded to glass slides. At this stage, devices were stored at room temperature, and then autoclaved for sterilization within 24 h prior to use. For studies integrating SI vasculature under SI epithelial monolayers (two chamber design), molds were fabricated according to specifications as shown, and molds were used to make PDMS devices as described above. The bottom chambers were plasma bonded to glass slides. Purchased cell culture membranes (CLS3428-24EA, Corning®; Corning, NY) were prepared by soaking them in 5% APTES (440140, Sigma-Aldrich®; St. Louis, MO) diluted in dH20 at 80 °C for 20 min similar to previously described^58^, drying them, and cutting them to size manually. Membranes were then plasma bonded to the bottom chamber mold. Finally, the top chamber was plasma bonded to the membrane-attached bottom chamber. At this stage, devices were stored at room temperature, and sterilized by UV light prior to use. A total of six pipette tips were used for vasculature only devices (two per media line, and one on either side of the culture chamber). An additional two pipette tips (eight total) were used when an upper chamber was added for GOC studies.

### Cell culture

Endothelial Colony Forming Cell-derived Endothelial Cells (ECFC-ECs, or ECs) were isolated from umbilical cord blood as previously described^59^ and used between passages 4 and 8 when passaged at a 1:2 ratio. ECs were expanded on tissue culture flasks pre-treated with 1% gelatin (G1890, Sigma) diluted in sterile de-ionized H2O. ECs were cultured in Endothelial Basal Medium (EBM) with EGM-2 BulletKit Supplementation (CC-3162, Lonza; Basel, Switzerland). Immunofluorescent staining (see method below) with anti-CD31 and anti-*ACTA2* was performed to confirm EC identity (CD31 positive, *ACTA2* negative) and exclude the presence of contaminating myofibroblast or pericyte populations (both *ACTA2* positive), prior to their use in culture.

Intestinal subepithelial myofibroblasts (ISEMFs) were isolated from patient surgical or biopsy specimens according to Washington University protocol 201504100 (Dr. Deborah Rubin) and used between passages 5 and 10. ISEMFs were expanded in DMEM, high glucose (11965–0840, Gibco; Waltham, MA) supplemented with 1% v/v MEM Nonessential Amino Acids (25-025-CI, Corning Cellgro), 1% v/v Glutamax (35050–061, Life Technologies; Carlsbad, CA), 10% v/v Fetal Bovine Serum (10438–026, Life Technologies), and 1% v/v Antibiotic-Antimycotic (15240062, Gibco). Prior to use, ISEMFs were evaluated using RT-qPCR (see method below) for high relative expression of *VIM* and *ACTA2*, and low *DES* expression, to confirm myofibroblast identity. The ability of ISEMFs to induce EC capillary formation was confirmed using three ISEMF donors.

For studies of SI angiogenesis, co-culture of ECs and ISEMFs in microfluidic devices was performed by re-suspending pelleted ECs and ISEMFs at a 1:1 v/v density in fibrinogen (F8630–10G, Sigma) diluted to 10 mg/ml in Dulbecco’s phosphate-buffered saline with calcium and magnesium (14040133, Gibco). Immediately prior to use, thrombin (T4648, Sigma) was added at 3 U/mL to resuspended cells in fibrinogen, yielding semi-solid fibrin gel. The central chamber of the microfluidic device was loaded with cells in fibrin. After loading, devices were incubated at 37° for 30 minutes to allow fibrin to solidify. EBM supplemented with EGM-2 BulletKit growth factors, as indicated by experimental condition, was then introduced into the top and bottom media lines, as shown, and changed daily. Cultures were performed in a 5% oxygen, 5% CO_2_ incubator with the exception of Figs. 1F, 2, 3C, 4A,B (21% oxygen, 5% CO_2_).

For studies with HIECs, ileal epithelium was harvested from patient surgical or biopsy specimens according to Washington University protocol 201404112 (Dr. Matthew Ciorba) and expanded as enteroids according to previously published methods^60,61^. GOC devices as shown in Fig. 4C were loaded with vasculature in the lower chamber on day 0 of the experiment as described above. On day 3, enteroids were dissociated into small cell aggregates, and loaded into the upper chamber. Static conditions were maintained overnight to facilitate cell attachment. Prior to loading HIECs, membranes were pre-treated with collagen IV (C5533, Sigma) at 34 µg/ml. L-WRN media with added Y-27632 dihydrochloride (125410, Tocris Bioscience; Bristol, United Kingdom) at 10 μM and SB 431452 (161410, Tocris Bioscience) at 10 μM was used in the upper chamber, while EGM-2 media was used in the lower chamber for the duration of the experiment. 24 h prior to fixation, the concentration of L-WRN in the upper chamber media was decreased, and Y-27632 and SB 431452 were removed, to facilitate cell differentiation.

All experiments involving human tissues were carried out in accordance with relevant guidelines and regulations. All experimental protocols were approved by the Washington University Human Research Protection Office (HRPO). Informed consent was obtained from all subjects. While each patient tissue type was obtained under its own protocol (as indicated), all aspects of the study—including co-culture of multiple deidentified patient tissues— were performed according to Washington University protocol 201610114.

### Transwell assays and TEER measurements

Transwell assays were performed using HIECs that had been expanded, as above. Monolayers were generated with enteroids as described previously, with slight modification^62^. Transwell inserts with tissue culture treated PET membranes with 0.4 μm pore size (3450, Corning®) were pre-treated with collagen IV as above for 1 h at 37 °C. Enteroids were trypsinized, passed through a 70 μm filter, and then resuspended in 50% L-WRN at approximately 2.0 × 10^4^ cell fragments per monolayer. Transwell units were placed into 24-well plates containing medium alone, ECs, ISEMFs, ECs with ISEMFs, or ECs with ISEMFs suspended in fibrin (with cell density at plating matched across all conditions). Medium in the top and bottom chambers were L-WRN and EGM-2, respectively, both of which were changed every other day. TEER was measured using an epithelial voltohmmeter (World Precision Instruments, Sarasota, FL).

### EGFR inhibition

EGFR inhibition was performed by adding receptor tyrosine kinase inhibitor Erlotinib (S7786, Selleckchem; Houston, TX), resuspended in DMSO, to culture media, achieving a 5 or 10 μM final concentration. DMSO at equal volume was used for vehicle control.

### Lentiviral fluorescent protein transfections

Azurite fluorescent protein lentiviral plasmid was generated by transducing HEK293T using three helper plasmid vectors (pMDLg-pRRE (Plasmid #12251), pRSV-Rev (Plasmid #12253), pMD2.G (Plasmid #12259), all Addgene; Cambridge, MA) and a third-generation lentiviral vector (Addgene pLV-Azurite, Plasmid #36086) in accordance with Washington University protocol 2947. Plasmid titers were then applied to ECs cultured in EGM-2 media with polybrene [5ug/ml] for 24–48 hours, yielding a ~60% stable transfection rate as confirmed by fluorescent microscopy.

### RT-qPCR

RNA isolation from cultured cells was performed per manufacturer instructions using the Total RNA Purification Kit (37500, Norgen; Thorold, ON). RNA concentration was measured using a NanoDrop Spectrophotometer (ND-1000; NanoDrop Technologies, Wilmington, DE). Relative mRNA expression was obtained using the Applied Biosystems 7500 Fast Real-Time PCR system (Applied Biosystems; Foster City, CA). Primer probes used were Hs00426835_m1 (*ACTA2*), Hs00958111_m1 (*VIM*), and Hs00157258_m1 (*DES*), Hs00911700_m1 (*KDR*), Hs00169795_m1 (*VWF*), Hs00174344_m1 (*CDH5*), and Hs02786624_g1 (*GAPDH*, internal control), all from Applied Biosystems.

### Immunohistochemistry

At the designated experimental endpoint, devices were fixed with 10% neutral buffered formalin, blocked with 2% BSA in DPBS, and stained with the following antibodies as indicated. Primary antibodies were: anti-CD31 (M0823, Dako; Santa Clara, CA; used at 1:200), anti-*ACTA2* (ab7817, Abcam; Cambridge, MA; used at 1:200 for EC identity verification during isolation), anti-*ACTA2* (ab202295, Abcam, used at 1:250 for ISEMF identity verification), anti-MUC2 (SC15334, Santa Cruz Biotechnology; Dallas, TX; used at 1:200), anti-KI-67 (9129 T,Cell Signaling Technology; Danvers, MA; used at 1:400), anti-CC3 (9664 S, Cell Signaling Technology, used at 1:400), and anti-UEA-1 (RL-1062, Vector Laboratories; Burlingame, CA; used at 1:500). Secondary antibodies were: goat anti-rabbit Alexa Fluor 555 (A21428, Invitrogen/ThermoFischer Scientific; Waltham, MA; used at 1:500), and rabbit anti-mouse Alexa Fluor 488 (A27023, Invitrogen/ThermoFischer Scientific, used at 1:500). 4′,6-Diamidino-2-Phenylindole, Dihydrochloride (DAPI) was used at 300 nM (D1306, Invitrogen).

### Microscopy

Device imaging was performed using inverted fluorescent microscopy (Olympus IX83 microscope, Olympus; Tokyo, Japan) with MetaMorph software (Molecular Devices; San Jose, CA), with the following exceptions: Growth factor titration, KI-67, CC3, and UEA-1 experiments were imaged using a Zeiss Axio Observer.Z1 microscope with a Zeiss Axiocam 506 mono camera (Carl Zeiss AG; Oberkochen, Germany). The integrated GOC model was imaged using a Zeiss LSM 880 II Airyscan FAST confocal microscope with the accompanying Zeiss Zen Microscope software, as was the EC only bead perfusion study. DAPI staining of HIEC monolayers on Transwells was imaged using a Leica SP8X tandem scanning confocal microscope with a white light laser 40x oil objective, with LASX by Leica Microsystems software (Leica Microsystems; Wetzlar, Germany).

### Quantifying capillary development

For all experiments, ECs were either transfected to express fluorescent protein (as above), or devices were stained for ECs using anti-CD31 antibody and a fluorescently labeled secondary antibody (as above). For each experiment, the same method was used in both EC only and EC + ISEMF conditions (as indicated in the text). Images were captured using fluorescent microscopy, allowing visualization of capillary networks based on EC fluorescence. “Total vessel length” was measured using AngioTool (https://ccrod.cancer.gov/confluence/display/ROB2/Home)^24^. Vessel maps, as automated by the program, were visually validated by a blinded reviewer to ensure accuracy. Total vessel length was calculated by summing the measurements of all vessel lengths within a high-powered field (hpf). Vessel lengths were measured as the linear distances between vessel junctions (or branch points). “Junction number” was also measured using Angiotool, and visually validated by a blinded reviewer to ensure accurate vessel mapping by the program. When discrepancies between automated mapping and visual review occurred, manual measurements were preferentially utilized. “Endothelial cell expansion” was quantified by either binary image analysis of EC fluorescence using Image J (National Institutes of Health; Bethesda, MD), or Angiotool analysis, and visual validation. For Image J binary analysis, briefly, a threshold was applied to distinguish fluorescence from background to generate a black and white image. Images were then measured for percent fluorescence as a ratio of black to white pixels. “Average vessel diameter” was measured manually by a blinded reviewer, using Image J to determine vessel diameters. All measurements were calibrated according to microscope and magnification settings, and averaged for each image. All images (non-overlapping) were averaged per device, and there were at least 2 devices per condition.

### Statistical analysis

Statistical analysis was completed using GraphPad Prism 7.0 (GraphPad Software; La Jolla, CA). Differences in quantifiable variables (above) were analyzed using unpaired Student’s *t*-tests or one-way ANOVA with Tukey’s multiple comparisons test, in the case of multiple groups. Number of variables are indicated in the figure legends. Graphs with error bars represent mean +/− SD. *P* < 0.05 was considered significant with *P < 0.05, **P < 0.01, ***P < 0.001, ****P < 0.0001.

## Data Availability

All data generated or analyzed during this study are included in this published article.
